# Angiotensin II type 2 receptor prevents extracellular matrix accumulation in human peritoneal mesothelial cell by ameliorating lipid disorder via LOX‐1 suppression

**DOI:** 10.1080/0886022X.2022.2133729

**Published:** 2022-10-13

**Authors:** Jing Liu, Bo Jin, Jian Lu, Yuan Feng, Nan Li, Cheng Wan, Qing-Yan Zhang, Chun-Ming Jiang

**Affiliations:** Institute of Nephrology, Nanjing Drum Tower Hospital, The Affiliated Hospital of Nanjing University Medical School, Nanjing, China

**Keywords:** High glucose, angiotensin II type 2 receptor, lectin-like oxidized lipoprotein receptor-1, extracellular matrix accumulation

## Abstract

Evidence suggests that intracellular angiotensin II type 1 receptor (AT1) contributes to peritoneal fibrosis (PF) under high glucose (HG)-based dialysates. It is generally believed that AT2 antagonisticly affects AT1 function. The aim of this study was to explore whether AT2 activation is beneficial for attenuating human peritoneal mesothelial cell (HPMC) injury due to HG. We treated a HPMC line with HG to induce extracellular matrix (ECM) formation. AT2 was increased and blocked using CGP42112A and AT2 siRNA. Lipid deposition was detected, signaling molecules associated with lectin-like oxidized lipoprotein receptor-1 (LOX-1) and ECM proteins were evaluated by real-time PCR and western blot. The results showed that HG led to AT2 inhibition in HPMCs, inhibition of AT2 further aggravated the expression of ECM proteins, including α-smooth muscle actin, fibroblast specific protein-1 and collagen I, while AT2 decreased the expression of ECM proteins, even during HG stimulation. Interestingly, there was a parallel change in lipid accumulation and ECM formation when AT2 was increased or depressed. Moreover, AT2-mediated decreased ECM production was associated with reduced lipid accumulation in HPMCs and depended on the downregulation of LOX-1. Further analysis showed that HG increased oxidized low-density lipoprotein (ox-LDL) deposition in HPMCs concomitant with an enhanced expression of ECM components, whereas blocking LOX-1 reversed ox-LDL deposition even in the presence of HG. This effect was also accompanied by the remission of ECM accumulation. Our results suggested that AT2 prevented ECM formation in HG-stimulated HPMCs by ameliorating lipid *via* LOX‐1 suppression.

## Introduction

Peritoneal dialysis (PD) is the first line treatment and a successful home-based dialysis modality for patients with end-stage renal disease (ESRD) [1]. Unfortunately, the peritoneal membrane frequently exhibits structural damage following long-term dialysis due to exposure to nonphysiological PD solution (PDS) with high glucose (HG) and low pH [2–4]. Many studies have suggested that human peritoneal mesothelial cells (HPMCs) synthesize matrix proteins, including α-smooth muscle actin (α-SMA) and fibroblast specific protein-1 (FSP1), together with collagen I, which are increased by HG. This synthetization leads to peritoneal fibrosis (PF) characterized by accumulation of extracellular matrix (ECM) in the submesothelial layer [5–7]. However, the pathogenic mechanisms underlying the deposition of ECM in HPMCs treated with HG-PDS have yet to be fully elucidated.

Angiotensin II (Ang II) is known to mediate fibrosis in a number of tissues, such as the peritoneum, vasculature, heart, kidney, and pharmacological inhibitors of the Ang II system, and is commonly used to alleviate this effect. Ang II exerts its effects by activating its receptors, primarily Ang II type 1 receptor (AT1) and AT2 [8]. Recent studies have proven that activation of AT2 exerts protective effects in many diseases. Terenzi et al. demonstrated that AT2 is strongly expressed in key effector cells of rheumatoid synovitis, and the activation of AT2 with a specific agonist CGP42112A may significantly reduce their proinflammatory and aggressive behavior. Therefore, AT2 agonism might represent a novel therapeutic strategy for patients with rheumatoid arthritis [9]. Sharma et al. showed that NP-6A4, a peptide agonist of AT2, has specific anti-inflammatory and vascular protective effects. Mechanistically, NP-6A4-AT2 signaling improved aortic distensibility by increasing the production of nitric oxide and inhibiting the osteopontin-matrix metalloprotease pathway [10]. Micakovic et al. reported that in the early stages of diabetes mellitus, AT2 overexpression in tubular epithelial cells alleviated all diabetes-induced renal changes, including a drop in mitochondrial bioenergetic efficiency, a rise in mitochondrial superoxide production, metabolic reprogramming, and increased proliferation. Thus, AT2 translocates to mitochondria and can contribute to renoprotective effects at early stages of diabetes [11]. It is unclear whether AT2 activation is beneficial in attenuating peritoneal damage due to HG stimulation.

Our previous study demonstrated that HG stimulates intracellular Ang II/AT1 signaling to induce disruption of low-density lipoprotein receptor (LDLr) negative feedback regulation. Subsequently, this dysregulation led to lipid deposition in HPMCs, thereby promoting ECM production [12]. To our knowledge, AT2 is thought to antagonisticly affect AT1 function, AT2 has been proven to improve lipid metabolism and prevent adiposity [13]. Sourashish et al. demonstrated that pharmacological activation of AT2 prevented high-fat diet-induced adiposity, dyslipidemia and inflammation and insulin resistance. The results indicate AT2 as a potential therapeutic approach for controlling obesity and obesity-associated disorders [14]. Recent studies have shown that there is a close relationship between lectin-like oxidized low-density lipoprotein scavenger receptor-1 (LOX-1) and AT2 [15,16]. LOX-1 is one of the important receptors responsible for binding, internalizing and degrading oxidized LDL (ox-LDL) [17]. The activation of LOX-1 is known to be connected with some pathophysiological events, including endothelial injury and dysfunction, fibroblast growth, and vascular smooth muscle cell hypertrophy. Many of these pathologic changes are involved in atherosclerosis [18], hypertension [19], and myocardial ischemia and remodeling [20]. Nevertheless, whether the decrease in AT2 levels facilitates the disruption of lipid homeostasis by LOX-1 and mediates peritoneal injury has not been explored.

To address the issue in this present study, we utilized an *in vitro* model to explore the protective effect of AT2 on HG-induced HPMC injury and the underlying molecular mechanism.

## Materials and methods

### Cell culture and stimulation

HPMC (HmrSV5) was purchased from Shanghai LMAI Biotech (L547). The cells were cultured in low glucose Dulbecco’s Modified Eagle’s Medium (DMEM; Gibco,10567-014) containing 10% fetal bovine serum (Gibco, 16000044). HPMCs were starved in serum-free DMEM containing 0.2% bovine serum albumin (BSA) (Sigma, Poole, Dorset, UK) for 24 h before the experiment. And the cells subsequently stimulated with HG, or CGP-42112A (Sigma, 127060-75-7).

### siRNA transfection

HPMCs were cultured and transiently transfected with AT2 siRNA (sc-29752), LOX-1 siRNA (sc-40185), negative control siRNA (sc-36869) and normal control siRNA (sc-29528) following the manufacturer’s protocol from Santa Cruz Biotechnology. Before seeding in a well containing 900 µL of serum free DMEM medium, 5 µL of AT2 siRNA, 5 µL of LOX-1 siRNA and 6 µL of siRNA transfection reagent were mixed with 100 μL of siRNA transfection medium (sc-36868) at room temperature for 30 min.

### Enzyme-linked immunosorbent assay (ELISA)

The ox-LDL ELISA kit was purchased from Shanghai Jianglai Biotech (JL47913). Samples and standards were pipetted into the antibody pre-coated microplate and incubated for 2 h at room temperature, avidin conjugated horseradish peroxidase and biotin-antibody were added into the wells and incubated for 1 h at room temperature. The optical density at 450 nm was read. The density of ox-LDL was calculated in accordance with the standard curve.

### Oil red O (ORO) staining

The lipid accumulation in HPMCs was observed by ORO staining. Briefly, the cells were harvested and washed three times with PBS, fixed with 1 h with a 5% formalin solution, stained with ORO (Sigma, O0625) for 30 min, and counterstained with hematoxylin (Sigma, H3136) for 5 min. Lastly, the cells were examined using light microscopy (×400).

### Filipin staining

HPMCs were fixed with 4% paraformaldehyde for 30 min and then stained in freshly prepared filipin solution for 30 min, followed by phenylenediamine/glycerol, and the slides were observed by laser confocal microscopy (×400).

### High-performance liquid chromatography (HPLC)

HPMCs were collected and lipid was extracted by adding 1 mL chloroform/methanol (2:1). The lipid phase was harvested, dried in a vacuum, and then dissolved in 2-propanol containing 10% Triton X-100. The contents of total and free cholesterol in each sample were detected *via* a standard curve normalized against total cell protein. The cholesterol ester concentration was calculated by subtracting the amount of free cholesterol from the total cholesterol.

### Real-time polymerase chain reaction (real-time PCR)

Total RNA was isolated from cultured HPMCs using the guanidinium-phenol-chloroform method. Then total RNA (500 ng) was reverse transcribed into cDNA using PrimeScript Reverse Transcription Reagent (TaKaRa, Japan). Real-time PCR was performed in an ABI7300 Sequence Detection System using SYBR green Gene Expression Assay (TaKaRa, Japan). All primers were designed using the Primer Express Software version 2.0 and shown in Table 1. The relative amount of mRNA was calculated by the comparative threshold cycle (2^−ΔΔCt^) method.

**Table 1.** Human TaqMan primers for real-time PCR.

Gene Primer	Sequences
AT2	Sense: 5′GACTGGCTCTTTGGACCTGTGAT 3′
	Antisense: 5′TTTGAGACAGAAAGGGGTAGATGAC 3′
α-SMA	Sense: 5′ACCCACAATGTCCCCATCTA3’
	Antisense: 5′GAAGGAATAGCCACGCTCAG3’
FSP-1	Sense: 5′GATGAGCAACTTGGACAGCA3’
	Antisense: 5′CTTCCTGGGCTGCTTATCTG3’
Collagen I	Sense: 5′CTGCAAGAACAGCATTGCAT 3′
	Antisense: 5′ GGCGTGATGGCTTATTTGTT3’
LDLr	Sense: 5′GCGAAAGAAACGAGTTCCAG3’
	Antisense: 5′TGACAGACAAGCACGTCTCC3’
LOX-1	Sense: TTGCCTGGGATTAGTAGTGACC
	Antisense: GCTTGCTCTTGTGTTAGGAGGT
β-actin	Sense: 5′CTACCTCATGAAGATCCTCACCGA 3′
	Antisense: 5′TTCTCCTTAATGTCACGCACGATT 3′

### Western blot analysis

Exact amount of protein extracts from HPMCs were denatured and resolved by electrophoresis. The membranes were incubated overnight at 4 °C with primary antibodies for AT2 (ab254561), α-SMA (ab7817), FSP-1 (ab197896), collagen I (sc-25974), LOX-1 (ab60178) and LDLr (sc-11824) followed by horseradish peroxidase-conjugated secondary antibodies for 2 h. Finally, the bands were examined using an enhanced chemiluminescence kit (Amersham Biosciences, UK). Relative expression levels were measured by normalization against glyceraldehyde-3phosphate dehydrogenase (GAPDH).

### Statistical analysis

Data are expressed as the mean ± standard deviation (SD) and were analyzed with SPSS 19.0 software. Student’s t-test was used for statistical significance analysis between two groups, and one-way ANOVA was performed to compare continuous variables among groups where appropriate. P-value less than 0.05 was considered statistically significant.

## Result

### HG inhibits AT2 in HPMCs

**Figure 1.**
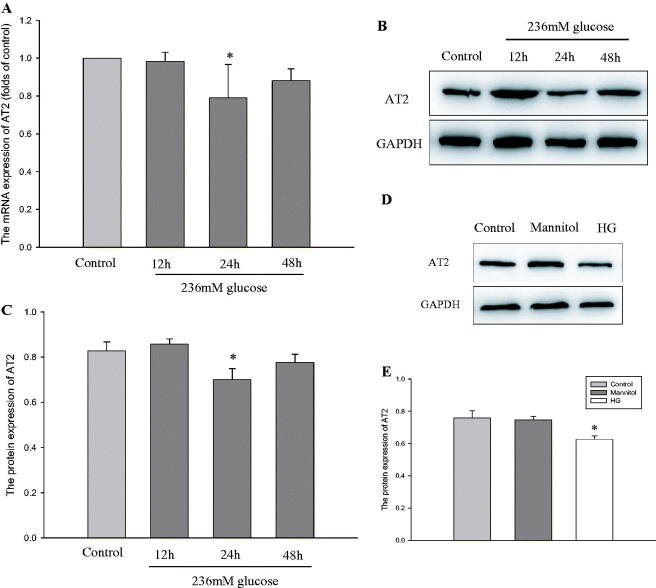
HPMCs were exposed to serum-free medium (control) or serum-free medium containing 236 mM glucose (HG) for 12 h, 24 h and 48 h. (A) Real-time PCR for the mRNA level of AT2 in HPMCs. β-actin served as the housekeeping gene. The results represent the mean ± SD from five experiments. *p < 0.05 *vs.* control. (B and C) The protein level of AT2 was determined by western blot analyses. The histogram shows the mean ± SD of the densitometric scans of the protein bands from five experiments following normalization by comparison with GAPDH. *p < 0.01 *vs.* control. (D and E) HPMCs were exposed to serum-free medium (control) or serum-free medium containing 236 mM mannitol (mannitol) or serum-free medium containing 236 mM glucose (HG) for 24 h. The protein level of AT2 was determined by western blot analyses. The histogram shows the mean ± SD of the densitometric scans of the protein bands from five experiments following normalization by comparison with GAPDH. *p < 0.01 *vs.* control.

To observe the effect of HG on AT2, HPMCs were stimulated with 236 mM glucose for 12 h, 24 h and 48 h. Results showed that compared with the control, HG significantly decreased the mRNA and protein level of AT2 in HPMCs for 24 h intervention (Figure 1(A–C)). In order to exclude the effect of osmolality, mannitol (236 mM) was used as an osmotic pressure control. The results showed that compared with the control, 236 mM glucose decreased the protein level of AT2 at 24 h, whereas no such effect was observed in mannitol group (Figure 1(D,E)). Based on the data, we finally stimulated HPMC with HG for 24 h for the rest of the experiment.

### AT2 attenuates ECM accumulation induced by HG in HPMCs

**Figure 2.**
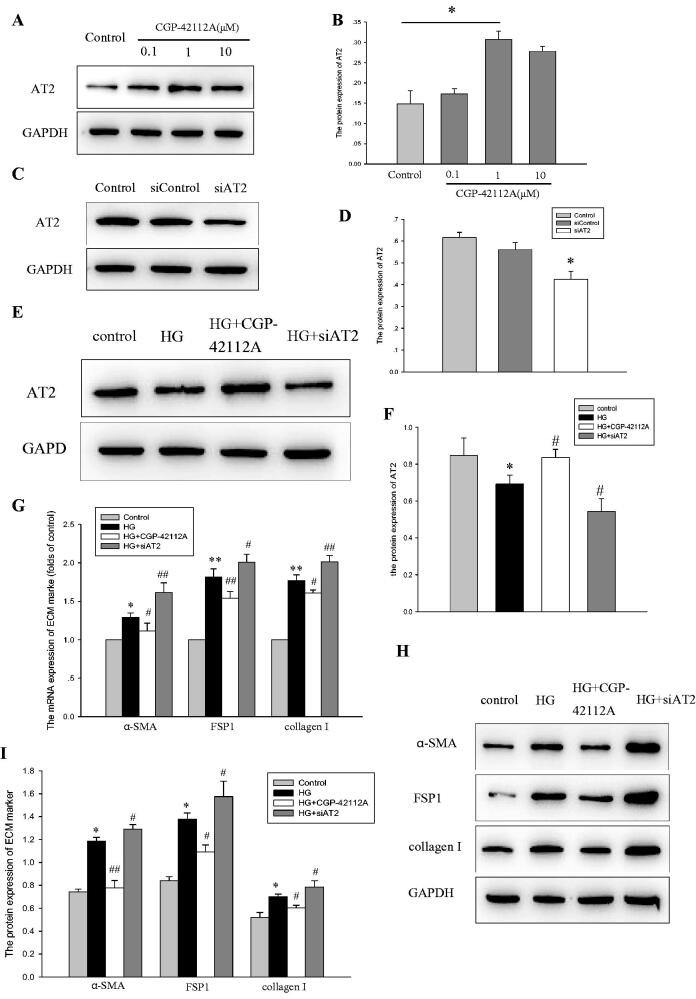
(A,B) HPMCs were treated with serum-free medium (Control) or in serum-free medium containing CGP-42112A (0.1, 1, and 10 µM) for 24 h. The protein level of AT2 was examined by western blot analyses. The histogram represents the mean ± SD of the densitometric scans of protein bands from five experiments, normalized by comparison with GAPDH. *p < 0.001*vs* control. (C,D) HPMCs were transfected with empty vector (Control), negative control siRNA (siControl), or AT2 siRNA (siAT2). The protein level of AT2 was examined by western blot analyses. The histogram represents the mean ± SD of the densitometric scans of protein bands from five experiments, normalized by comparison with GAPDH. *p < 0.05 *vs.* control. (E-I) HPMCs were exposed to serum-free medium (Control) or in serum-free medium containing 236 mM glucose (HG), 236 mM glucose plus 1 µM CGP-42112A (HG + CGP-42112A), or 236 mM glucose plus AT2 siRNA (HG + siAT2) for 24 h. (E and F) The protein level of AT2 was determined by western blot analyses. The histogram shows the mean ± SD of the densitometric scans of the protein bands from five experiments following normalization by comparison with GAPDH. *p < 0.05 *vs.* control, ^#^*p* < 0.05 *vs.* HG group. (G) Real-time PCR for the mRNA expression of α-SMA, FSP1 and collagen I in HPMCs. β-actin served as the housekeeping gene. The results represent the mean ± SD from five experiments. *p < 0.*01 vs.* control, **p < 0.001 *vs.* control, ^#^*p* < 0.05 *vs.* HG group, ^##^*p* < 0.01 *vs.* HG group. (H and I) The protein levels of α-SMA, FSP1 and collagen I were determined by western blot analyses. The histogram shows the mean ± SD of the densitometric scans of the protein bands from five experiments following normalization by comparison with GAPDH. *p < 0.001 *vs.* control, ^#^*p* < 0.05 *vs.* HG group, ^##^*p* < 0.001 *vs.* HG group.

We quantified the expression of critical proteins of the ECM, including α-SMA, FSP1 and collagen I. Consistent with our previous studies, the protein expressions of α-SMA, FSP1 and collagen I were increased in the HG group. To further determine the role of AT2 on HG-induced ECM, HPMCs were treated with the specific AT2 agonist CGP-42112A. As shown in Figure 2(A,B), 1 µM CGP-42112A significantly increased the level of AT2 at 24 h. Based on these data, we chose the dose for subsequent experiments in the study. Additionally, we treated the HPMCs with AT2 siRNA to inhibit the AT2 (Figure 2(C,D)). Next, we observed the protein level of AT2 treated with CGP-42112A or AT2 siRNA under HG, and found that when compared with the HG group, CGP-42112A increased the AT2 amount, but AT2 siRNA further reduced the AT2 amount (Figure 2(E,F)). Further analysis suggested that CGP-42112A prevented the protein expression of ECM markers even in the presence of HG environment. In contrast, the inhibition of AT2 with siRNA assay enhanced ECM accumulation in HPMCs (Figure 2(G–I)). The data implied that AT2 played a preventive effect on ECM accumulation in HPMCs stimulated with HG.

### AT2 suppresses lipid deposition induced by HG in HPMCs

**Figure 3.**
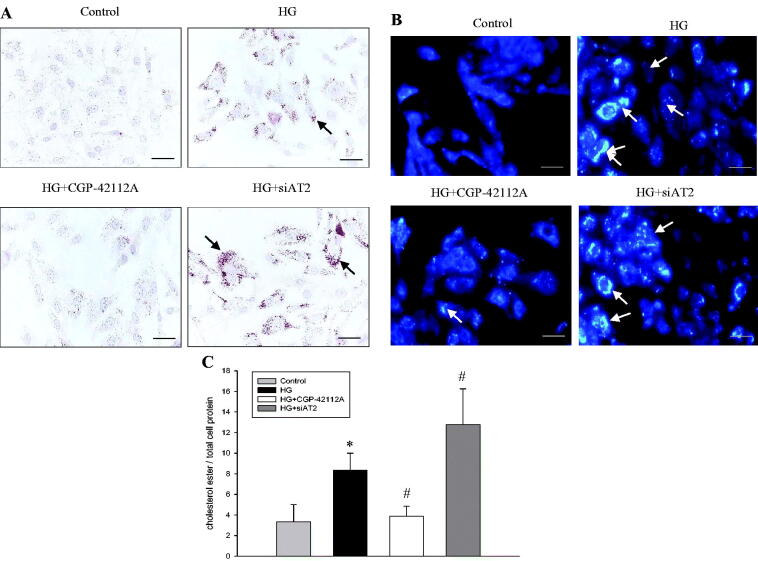
HPMCs were exposed to serum-free medium (control), serum-free medium containing 236 mM glucose (HG), 236 mM glucose plus 1 µM CGP-42112A (HG + CGP-42112A), or 236 mM glucose plus AT2 siRNA (HG + siAT2) for 24 h. **(A)** HPMCs were examined for lipid inclusion by ORO staining, and the positive areas were stained red as shown by the black arrow (original magnification ×400, the size of the bar = 50µm). **(B)** HPMCs were examined for lipid inclusion by filipin staining, and the positive areas were stained bright blue as shown by the white arrow (original magnification ×400, the size of the bar = 50µm). **(C)** The concentration of cholesterol ester in HPMCs was measured as described in the Materials and Methods. Values are the mean ± SD of duplicate wells from five experiments. *p < 0.01 *vs.* control, *^#^p* < 0.05 *vs.* HG group.

We next observed the effects of AT2 on lipid deposition in HPMCs. Compared with the control, a significant enhancement in lipid deposition was observed in HG-treated HPMCs, as showed by ORO staining and filipin staining. The activation of AT2 using CGP-42112A decreased HG-stimulated lipid droplet deposition in HPMCs, while inhibition of AT2 aggravated this effect (Figure 3A-B). This result was consistent with the data from intracellular cholesterol quantitative assays as demonstrated by HPLC (Figure 3C). These findings suggested that HG induces lipid deposition in HPMCs, which can be reversed by the AT2.

### AT2 alleviates the dysregulation of LOX-1 induced by HG in HPMCs

**Figure 4.**
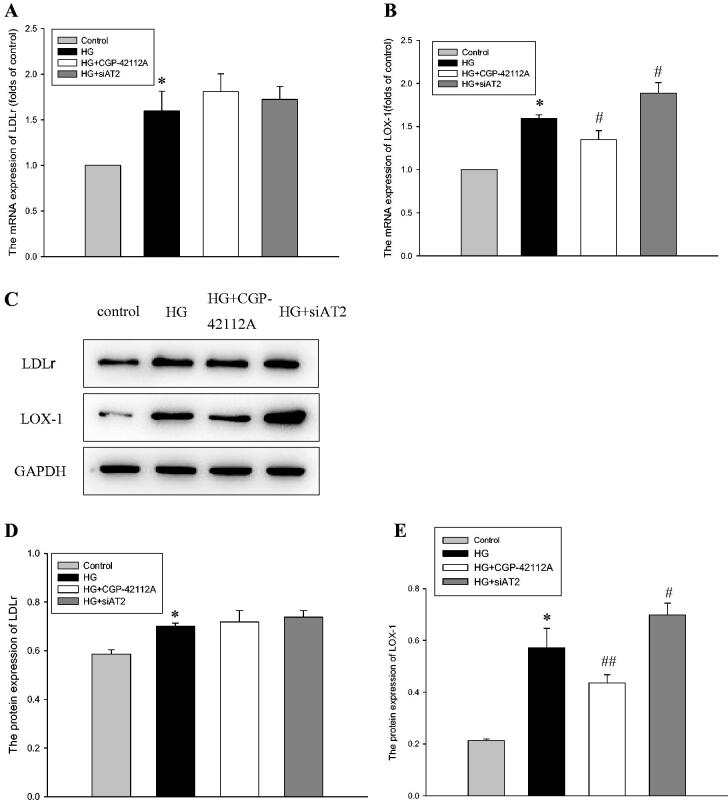
HPMCs were exposed to serum-free medium (control), serum-free medium containing 236 mM glucose (HG), 236 mM glucose plus 1 µM CGP-42112A (HG + CGP-42112A), or 236 mM glucose plus AT2 siRNA (HG + siAT2) for 24 h. (A) Real-time PCR for the mRNA expression of LDLr in HPMCs. β-actin served as the housekeeping gene. The results represent the mean ± SD from five experiments. *p < 0.01*vs* control. (B) Real-time PCR for the mRNA expression of LOX-1 in HPMCs. β-actin served as the housekeeping gene. The results represent the mean ± SD from five experiments. *p < 0.001*vs* control, ^#^*p* < 0.01 *vs.* HG group. (C-E) The protein levels of LDLr (D) and LOX-1 (E) were determined by western blot analyses. The histogram shows the mean ± SD of the densitometric scans of the protein bands from five experiments following normalization by comparison with GAPDH. *p < 0.*01 vs.* control, ^#^*p* < 0.05 *vs.* HG group, ^##^*p* < 0.01 *vs.* HG group.

To explore the potential mechanisms leading to lipid accumulation in HPMCs following HG intervention, we observed the expression of LDLr and LOX-1, which mediated the intracellular uptake of nLDL and ox-LDL, respectively. As shown in Figure 4, exposure of HPMCs to HG increased the mRNA and protein expression levels of LDLr and LOX-1. Cotreatment with CGP-42112A ameliorated the HG-induced change in LOX-1 expression but not LDLr expression. In addition, we also observed the inhibitory effect of AT2 on the expression of LDLr and LOX-1. The results showed that AT2 siRNA increased the expression of LOX-1 in HPMCs, while LDLr was not affected. These findings implied that the activation of AT2 ameliorated the dysregulation of LOX-1, which alleviated lipid deposition in HPMCs.

### Intracellular AT2 attenuates ECM accumulation by ameliorating the dysregulation of LOX-1 in HPMCs

**Figure 5.**
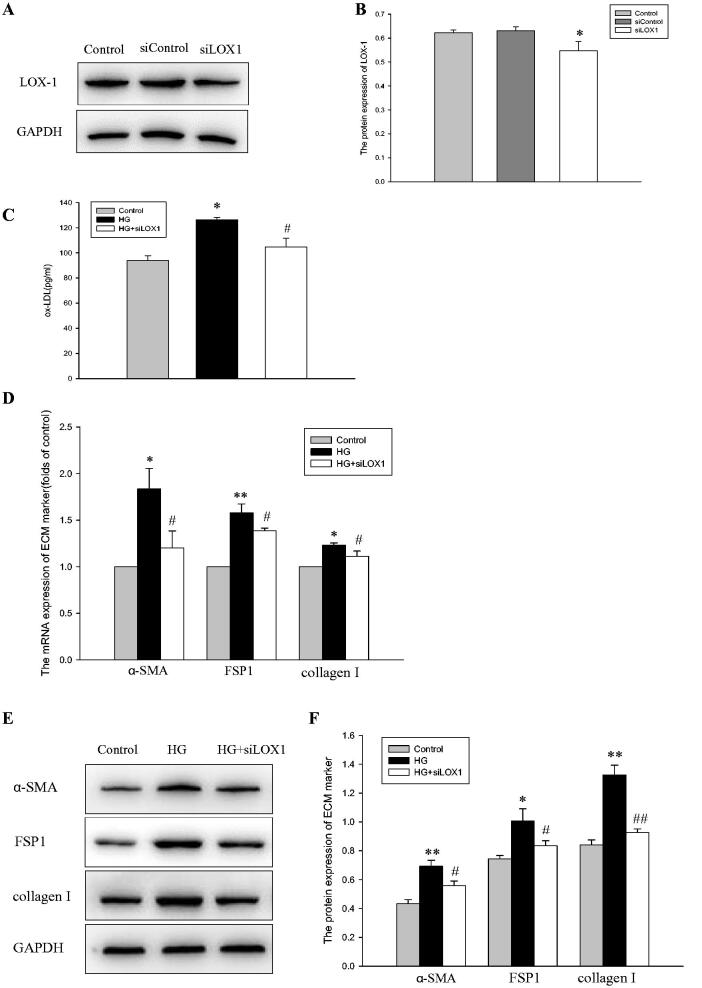
(A,B) HPMCs were transfected with empty vector (Control), negative control siRNA (siControl), or LOX-1 siRNA (siLOX1). The protein level of LOX-1 was examined by western blot analyses. The histogram represents the mean ± SD of the densitometric scans of protein bands from five experiments, normalized by comparison with GAPDH. *p < 0.01 *vs.* control. (C-F) HPMCs were exposed to serum-free medium (Control), serum-free medium containing 236 mM glucose (HG), or 236 mM glucose plus LOX-1 siRNA (HG + siLOX1) for 24 h. (C) The ox-LDL of the cell culture supernatant was measured by ELISA. *p < 0.01 *vs.* control, ^#^*p* < 0.05 *vs.* HG group. (D) Real-time PCR for the mRNA expression of α-SMA, FSP1 and collagen I in HPMCs. β-actin served as the housekeeping gene. The results represent the mean ± SD from five experiments. *p < 0.*01 vs.* control, **p < 0.001 *vs.* control, ^#^*p* < 0.01 *vs.* HG group. (E and F) The protein levels of α-SMA, FSP1 and collagen I were determined by western blot analyses. The histogram shows the mean ± SD of the densitometric scans of the protein bands from five experiments following normalization by comparison with GAPDH. *p < 0.*05 vs.* control, **p < 0.001 *vs.* control, ^#^*p* < 0.01 *vs.* HG group, ^##^*p* < 0.001 *vs.* HG group.

Finally, to investigate whether AT2-attenuated ECM accumulation in HPMCs *via* suppressing LOX-1, an RNA interference-mediated knockdown of LOX-1 was performed (Figure 5(A,B)). The results showed that HG mildly enhanced the level of ox-LDL; however, inhibition of LOX-1 restrained ox-LDL in HPMCs, as demonstrated by ELISA (Figure 5(C)). Further research showed that HG upregulated the mRNA and protein expression of α-SMA, FSP-1 and collagen I, while inactivation of LOX-1 decreased the expression of ECM proteins in HPMCs (Figure 5(D–F)). These results suggested that intracellular AT2 ameliorates the dysregulation of LOX-1, thus inhibiting ECM accumulation in HPMCs.

## Discussion

Long-term exposure to bioincompatible PDS, especially HG, plays an important role in ECM production of PMCs. HG has been shown to lead to fibrosis by inducing PMCs to lose the epithelial phenotype and obtain fibroblast-like characteristics, acquire matrix protein expression and ultimately ECM formation [21–23]. Our previous *in vivo* and *in vitro* study implicated a local renin angiotensin system in the peritoneum in the pathophysiology of PF under HG stimulation. Further results suggest that Ang-II/AT1R signaling contributes to intracellular lipid accumulation and that this dysregulation leads to ECM formation in HPMCs [12]. The role of AT2 in HG-induced peritoneal injury remains unclear.

Recently, several lines of evidence have demonstrated that activation of AT2 shows significant therapeutic benefits in diabetic nephropathy [11], rheumatoid arthritis [24], aortic stiffness [10]. In this study, we found that HG caused a decrease in the mRNA and protein level of AT2. In addition, higher expression of ECM components such as α-SMA, collagen I and FSP-1 were observed in cultured HPMCs exposed to HG. Furthermore, activation of AT2 using CGP-42112A overrode HG-stimulated ECM formation in HPMCs, while inhibition of AT2 with AT2 siRNA enhanced these effects when compared with HG. Therefore, these observations implied a protective role of AT2 in HPMC injury. However, the mechanisms responsible for the beneficial effects of AT2 have not yet been elucidated.

AT2 has been discussed as a target to test its role in lipid metabolism, and recent pharmacological studies have explicitly suggested that AT2 activation inhibits adiposity and obesity. For instance, AT2 agonist C21 administration in male mice reduced adiposity and improved lipid metabolism, likely by inhibiting lipid synthesis and enhancing lipid degradation. Further *in vitro* studies in freshly isolated adipocytes from mice showed that AT2 activation decreases fatty acid uptake through the nitric oxide/cyclic guanosine monophosphate pathway [25]. Matsushita et al. also demonstrated that pharmacological blockade of AT2 promotes adipogenesis in mesenchymal stem cells; moreover, this inhibitory effect is associated with wnt10b/β-catenin signaling [26]. Our results in Figure 3 showed that HG increased lipid accumulation in HPMCs, and this effect was mitigated by CGP-42112A but enhanced by AT2 siRNA. There was a parallel change in the expression of ECM components and intracellular lipid content when treated with HG or AT2 agonist or AT2 inhibitor. Thus, we assumed that the protective effect of AT2 on HPMC is closely related to decreased intracellular lipid deposition.

To explore the mechanism of action through which AT2 affects lipid deposition in HPMCs, we focused primarily on LDL cholesterol metabolism because LDL is the primary determinant of abnormal accumulation of cholesterol in diseased conditions. Similarly, biochemical modification of LDLs is a pathogenic hallmark in various diseases; notably, of all the lipid modifications discovered, ox-LDL has been identified as the primary risk factor for vascular inflammation, lipid accumulation and plaque formation [27]. Ox-LDL exerts its effect through some different receptors, the most important of which is LOX-1 [28]. As shown in Figure 4, we found that HG significantly upregulated LDLr and LOX-1 expression, and the high expression of LOX-1 was reduced with CGP-42112A but increased *via* AT2 siRNA. Interestingly, there was no obvious change in LDLr expression when used with an AT2 agonist or AT2 inhibitor. The results demonstrated that AT2 influenced LOX-1 signaling but not LDLr. Hu et al. found that AT2 overexpression could effectively prevent the enhancement of LOX-1 expression caused by hyperlipidemia, and LOX-1 has the potential to promote the accumulation of collagen in atherosclerotic plaques. The researchers showed that the pathogenesis of atherosclerosis may involve an interplay of LOX-1 and AT2 [15]. To further clarify whether anti-LOX-1 signaling-mediated decreased lipid accumulation in HPMCs by AT2 would mitigate pathologic peritoneal injury, LOX-1 siRNA was performed. HG promotes ox-LDL uptake by HPMCs, whereas deletion of LOX-1 overrode this effect. Additionally, LOX-1 siRNA decreased α-SMA, FSP1 and collagen I formation even in the presence of HG stimulation, which implied that AT2 ameliorates intracellular ox-LDL deposition and then mitigates HPMC injury through inhibition of the expression of LOX-1.

**Figure 6.**
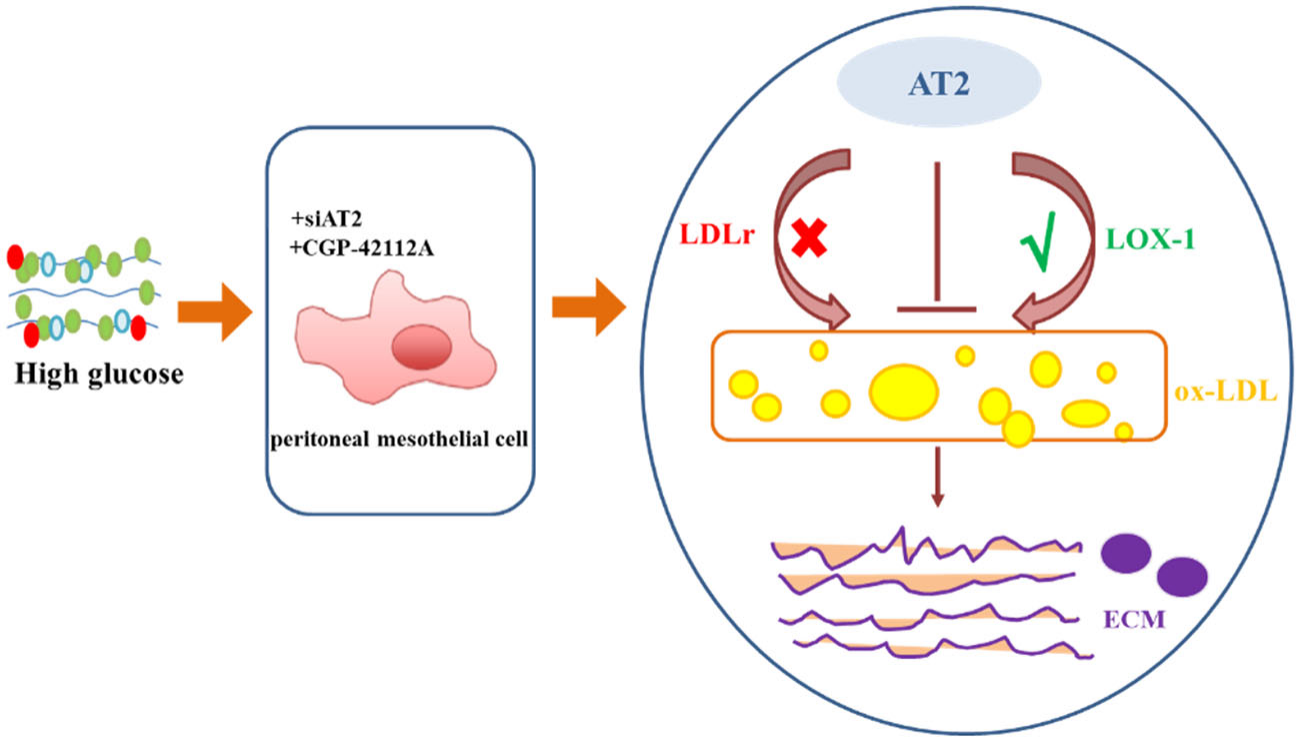
Schematic model of the protective mechanism of AT2 against HG-induced HPMC injury. AT2 significantly prevents HG-induced ECM accumulation in HPMCs, which manifests as decreased expression levels of α-SMA, FSP1 and collagen I. These effects are correlated with a decrease in the deposition of intracellular ox-LDL, which is mediated by the downregulation of LOX-1. Overall, intracellular AT2 exerts an obvious protective effect against HG-induced ECM accumulation in HPMC, which is partly due to the downregulation of LOX-1 mediating lower ox-LDL uptake in HPMCs.

Therefore, AT2 can improve intracellular cholesterol homeostasis through reduced expression of LOX-1 inhibiting ox-LDL uptake, consequently alleviating ECM accumulation in HPMC caused by HG (Figure 6). In summary, HG is a crucial risk factor for the progression of HPMC injury, and anti-LOX-1 *via* an AT2 agonist may reverse pathological damage and may be a new thought for the clinical treatment of PF.
